# Therapeutic Education Program for Children and Adolescents with chronic diseases - Letter to the Editor

**DOI:** 10.6061/clinics/2020/e2419

**Published:** 2020-11-10

**Authors:** Liz Avlasevicius Oliveira, Magda Maria Sales Carneiro-Sampaio, Angela Midori Matuhara, Louise Cominato, Regina Celia Turola Passos Juliani, Aurora Rosaria Pagliara Waetge

**Affiliations:** I Instituto da Crianca e do Adolescente (ICr), Hospital das Clinicas (HCFMUSP), Faculdade de Medicina, Universidade de Sao Paulo, Sao Paulo, SP, BR; II Faculdade Medicina (FMUSP), Universidade de Sao Paulo, Sao Paulo, SP, BR; III Departamento de Neonatologia, Faculdade de Medicina (FMUSP), Universidade de Sao Paulo, Sao Paulo, SP, BR; IV Unidade de Endocrinologia do Instituto da Crianca e do Adolescente (ICr), Hospital das Clinicas (HCFMUSP), Faculdade de Medicina, Universidade de Sao Paulo, Sao Paulo, SP, BR

To the Editor,

Health care often places patient assistance centered on prescriptive acts that frequently result in procedures effective only in the biomedical dimensions without considering the social, environmental, and circumstantial factors that affect the evolution of the health status of a person with a chronic disease (1).

Health care production involves promotion, prevention, recovery, surveillance, assistance, and rehabilitation. The line of care, therefore, involves robust management in order to better articulate interventions as the social determinants of the health-disease process (2) and instrumentalizing of the care agents to look at the subjectivity and singularity in order to orchestrate the health care exercise in its entirety. 

The care, in its integrality, should indispensably include teamwork improvement as it is the sum of specific actions of each professional from different nuclei with their knowledge and practices in the unique field of action for the construction of strategy within the institutional arrangements in order to operate the daily management of the micro politics of care that result in more mindful interventions (3).

The improvement of a type of medicine centered toward patient care instead of the disease necessarily includes the role of the medical personnel as well as the autonomy of the patients and their families. Kleinman (4) states that the disagreement between the doctor and patient regarding the disease, the process of becoming ill, and the goals to be achieved with the treatment have a negative impact on the results of the medical interventions.

Therapeutic education in health is a qualitative tool based on human resources for forming a health care system for professionals, patients, and their families. Since 2019, the Institute of Children and Adolescents of Hospital das Clínicas of the Faculty of Medicine of the University of Sao Paulo has promoted the implementation of the project “Therapeutic education for children and adolescents with chronic diseases” in three outpatient clinics, namely: Cystic Fibrosis Clinic (Pneumonology Unit), Bullous Epidermolysis (Pain and Palliative Care Unit), and Obesity (Endocrinology Unit). This project aims to include qualified health-care personnel in order to improve the autonomy of the patients and their families in the course of the treatment. The goal is to alleviate the burden on professionals, reduce costs on public health, as well as to integrate the health care offered to children and adolescents suffering from chronic diseases in a multi, inter, and transdisciplinary way.

In order to plan the execution of this project, monthly meetings have been held between residents and professionals who volunteered as participants in the preparation and alignment of the action plan.

Through the use of design thinking management tools, it was possible to plan the different stages of this project, as design techniques were used to solve old problems in an innovative way (5,6).

Initially, the itinerary of patients and their companions was monitored by the residents involved in the project in the three outpatient clinics, with the purpose of knowing their route within the institution and the difficulties they encountered. This phase had an observational component and it made possible at the same time to identify the limits and potentialities of the paths taken by the patients. In this stage it was also possible to identify issues with physical structure, team communication, and the understanding of the treatment.

After this stage, two structured questionnaires were prepared for both professionals working in the outpatient clinics and the patients / patient-companions. The objective was to better understand the health care experiences both offered and received, as well as the difficulties and advantages in following the therapeutic plan by both parties.

The questionnaires were sent to the participants via email, with electronic reminders within one month after the first sending. The data obtained were analyzed statistically and categorized to organize the results.

Considering the importance of offering comprehensive health care, the project, currently underway, in its next steps, aims to develop expanded therapeutic plans for each patient in the aforementioned outpatient clinics and operationalize the implementation of information systems that allow the monitoring of care, monitoring and evaluation of actions and services for health promotion and protection, disease prevention, diagnosis, treatment, rehabilitation, reduction of harm to health, in addition to the development of a prototype plan of action intended to offer higher quality assistance and working conditions for professionals.

We believe that the participation of residents in the execution of this project is essential in its effectiveness, in addition to contributing to the technical and academic training of these professionals.

Finally, it is worth highlighting the innovative character of this project, which is based on its potential to add subjective experiences to technical knowledge in order to offer health care in a way that transforms lives.

## AUTHOR CONTRIBUTIONS

All the authors contributed substantially to the conception and design of the study. All authors revised the work critically and approved the final version.
